# Response to Kounali *et al*.'s letter of response

**DOI:** 10.1017/S0950268819001584

**Published:** 2019-09-20

**Authors:** P. J. White, J. Lewis

**Affiliations:** 1 MRC Centre for Global Infectious Disease Analysis and NIHR Health Protection Research Unit in Modelling Methodology, Imperial College London, London, UK; 2 Modelling and Economics Unit, National Infection Service, Public Health England, London, UK

Authors of the paper by Kounali *et al*. have repeatedly made the same challenge to the validity of our model-based method for estimating chlamydia prevalence from surveillance data, to which we have repeatedly responded, with evidence [1, 2] – none of which they have acknowledged.

Kounali *et al*., a group which includes representation of England's National Chlamydia Screening Programme (NCSP), assert ‘It is not possible… to make inferences about CT prevalence, or changes… over time, without information on [reason for testing]’, yet despite the lack of such data NCSP states that ‘modelling suggests that the level of testing that has been achieved in England… will probably have resulted in reductions in prevalence’ [3]. Regarding Kounali *et al.*'s citations in support of their assertion, we have already responded to Soldan *et al*.'s letter [1, 2] and the commentary of Low and Smid [1], and we cited the paper by Miller in our original paper [4].

Estimating chlamydia incidence and prevalence from surveillance data is a subject of active debate, and methods have been described in several papers by ourselves and others [4–6]. We would direct readers to our original paper (and accompanying computer code) describing our model and its testing, validation, and sensitivity analysis considering unrecorded information, so they may evaluate our method for themselves [4].

The point on which we all agree is the importance of understanding testing behaviour and individuals' reasons for chlamydia testing. We hope that more-detailed surveillance data and population-based studies such as the fourth National Study of Sexual Attitudes and Lifestyles will provide the information required to better-understand patterns of chlamydia infection and optimise the effectiveness and cost-effectiveness of chlamydia control.
